# Ultrasound Risk Categories for Thyroid Nodules and Cytology Results: A Single Institution's Experience after the Adoption of the 2016 Update of Medical Guidelines by the American Association of Clinical Endocrinologists and Associazione Medici Endocrinologi

**DOI:** 10.1155/2017/8135415

**Published:** 2017-06-28

**Authors:** Roberto Negro, Gabriele Greco, Ermenegildo Colosimo

**Affiliations:** ^1^Division of Endocrinology, “V. Fazzi” Hospital, Piazza F. Muratore, 73100 Lecce, Italy; ^2^Division of Pathology, “V. Fazzi” Hospital, Piazza F. Muratore, 73100 Lecce, Italy

## Abstract

**Objectives:**

In 2016, the American Association of Clinical Endocrinologists (AACE) and Associazione Medici Endocrinologi (AME) released updated guidelines for the diagnosis and management of thyroid nodules. The aim of this study was to evaluate the AACE/AME recommendations for FNA in clinical practice, by comparing the (US) stratification risk and indications for FNA with cytologic results.

**Methods:**

From May to December 2016, we collected the cytologic results from FNAs of nodules that were classified using a three-tier US category system (low, intermediate, and high risk).

**Results:**

We obtained 859 FNAs from 598 patients: 341 (39.7%) from low, 489 (56.9%) from intermediate, and 29 (3.4%) from high risk nodules. Of these, 88.5% and 74.9% of low and intermediate risk nodules, respectively, were cytologically benign, whereas 84.6% of high risk nodules had a moderate-to-elevated risk of malignancy or were malignant. If FNAs had been limited to intermediate risk nodules >20 mm, we would have missed 13/17 (76.5%) nodules that had moderate-to-elevated risk of malignancy or were malignant (11/13 were malignant based on histology).

**Conclusions:**

A nonnegligible number of cytologically malignant nodules or nodules that were suspected to be malignant would be missed if intermediate US risk nodules <20 mm were not biopsied.

## 1. Introduction

Thyroid nodules are a common finding in endocrinology. The widespread use of ultrasound (US) (unrelated to suspected thyroid disease), CT scan, MRI, and 18F-fluorodeoxyglucose has tremendously increased the number of patients suffering from a disease that is often benign and asymptomatic [1–4]. The recognition of a thyroid nodule entails a complete assessment that includes functional status (based on thyroid-stimulating hormone), a dedicated thyroid US, and fine needle aspiration (FNA), when necessary [5–7]. The reason for these evaluations, other than discovering any possible dysfunction (often subclinical), is to rule out malignancy. The American Thyroid Association (ATA), the American Association of Clinical Endocrinologists (AACE), and Associazione Medici Endocrinologi (AME) released guidelines about the diagnosis and management of thyroid nodules [8–10]. The 2016 AACE/AME guidelines provided suggestions about US risk categories of malignancy for thyroid nodules and the consequent indications for FNA [11]. Briefly, thyroid nodules are stratified using a three-class system based on their risk of malignancy as low, intermediate, or high risk. The FNA is recommended for the following cases: “high US risk thyroid lesions ≥10 mm”; “intermediate US risk thyroid lesions >20 mm”; “low US risk thyroid lesions only when >20 mm and increasing in size or associated with a risk history and before thyroid surgery or minimally invasive ablation therapy”; “subcapsular or paratracheal lesions”; “suspicious lymph nodes or extrathyroid spread”; “positive personal or family history of thyroid cancer”; and “coexistent suspicious clinical findings (e.g., dysphonia).” In addition, “nodules with a major diameter <5 mm should be monitored, rather than biopsied” and “in nodules with a major diameter of 5–10 mm that are associated with suspicious US signs, either FNA or watchful waiting may be considered.” Moreover, six diagnostic classes are recommended for cytologic reports: “(1) nondiagnostic; (2) benign; (3) atypia or follicular lesion of undetermined significance (AUS/FLUS); (4) follicular neoplasm or lesion suspicious for follicular neoplasm (FN/SFN); (5) suspicious for malignancy; (6) malignant” [12]. The aim of the present study was to evaluate the 2016 AACE/AME suggestions by comparing the US stratification risk and recommendations for FNA with cytologic results and the consequent clinical strategies.

## 2. Methods

Beginning in May 2016 when the AACE/AME guidelines were released, we adopted the US risk stratification in patients referred to our division for thyroid nodules as follows: “*Class 1: low risk thyroid lesion*: mostly cystic (>50%) nodules with reverberating artifacts that are not associated with suspicious US signs; isoechoic spongiform nodules confluent; or regular halo;* Class 2: intermediate risk thyroid lesion*: slightly hypoechoic nodules (cf. surrounding thyroid tissue) and isoechoic nodules with ovoid-to-round shape and smooth or ill-defined margins; intranodular vascularization; elevated stiffness by elastography; macro- or continuous rim calcifications; or hyperechoic spots of uncertain significance;* Class 3: high risk thyroid lesion*: nodules with at least 1 of the following suspicious features: marked hypoechogenicity (cf. prethyroid muscles); spiculated or microlobulated margins; microcalcifications; taller-than-wide shape; evidence of extrathyroidal growth or pathologic adenopathy.” When a FNA was obtained, the report for the cytopathologist included the US risk categorization of the nodule. Then, we retrospectively evaluated the US risk categories and the cytologic reports of patients who underwent FNA from May to December 2016. In the present analysis, we included only patients with solid or predominantly solid nodules. Patients with subclinical or overt hyperthyroidism (i.e., those with autonomously functioning nodules) were excluded. The Italian consensus for the classification and reporting of thyroid cytology was adopted in 2014 and is currently used by our cytopathologists [13]. This is a six-category system, similar to that of the Bethesda System (TIR1: nondiagnostic = nondiagnostic; TIR2: nonmalignant = benign; TIR3A: low risk indeterminate lesion = AUS/FLUS; TIR3B: high risk indeterminate lesion = FN/SFN; TIR4: suspicious for malignancy = suspicious for malignancy; TIR5: malignant = malignant).

### 2.1. Statistical Analysis

For descriptive statistics, Statistical Package for Social Sciences (SPSS 15.0 for windows; SPSS Inc., Chicago, IL, USA) was used. The *P* values were measured using Student's *t*-test for continuous variables. In all analyses, *P* < 0.05 was considered statistically significant.

## 3. Results

There were 859 FNAs performed in 598 patients (493 females and 105 males), with 215/598 (36%) patients having more than one nodule biopsied. The mean age was 56.2 ± 13.6 years. The mean dimensions (mm) of nodules were as follows: anteroposterior: 14.3 ± 6.8, transverse: 14.2 ± 6.9, and longitudinal: 19.6 ± 9.5. The mean volume (mL) was 3.7 ± 7.1. The nodules were divided into the three US categories of risk as follows: 341 (39.7%) were low risk, 489 (56.9%) were intermediate risk, and 29 (3.4%) were high risk. Diameters (anteroposterior, transverse, and longitudinal) were smaller in high risk nodules versus intermediate and low risk. The volume did not significantly differ among the three categories (Table 1). When comparing nodules by size, those <10 mm were more frequently at high risk, whereas those >20 mm were more frequently at low risk. When comparing nodules from all three US categories of risk, nodules with a diameter of 10–20 mm were more frequently biopsied (Table 2).

Altogether, we obtained 83 TIR1 (9.7%), 605 TIR2 (70.4%), 129 TIR3A (15.0%), 19 TIR3B (2.2%), 6 TIR4 (0.7%), and 17 TIR5 (2.0%) nodules (TIR2 versus other results; *P* < 0.01). Inadequate samples were equally distributed among the low, intermediate, and high risk nodules. Most cytologic results from nodules in the low or intermediate risk category were benign (TIR2) (88.5% and 74.9%, resp.), whereas the majority (84.6%) of nodules in the high risk group had moderate-to-elevated risks of malignancy or were malignant (TIR3B/TIR4/TIR5) (Table 3). If the FNA had been limited to intermediate risk nodules >20 mm and if we had further excluded low risk nodules with any diameter, we would have missed all three TIR3B nodules in the low risk group (all benign by histology) and 13/17 (76.5%) nodules that had moderate-to-elevated risks of malignancy or were malignant (TIR3B/TIR4/TIR5) in the intermediate risk group (11/13 were malignant by histology). If we had chosen watchful waiting instead of FNA for high risk lesions <10 mm, we would have not received a cytologic confirmation of malignancy in 8/26 (30.8%) cases.

## 4. Discussion

Ruling out malignancy in thyroid nodules represents one of the major and most frequent problems in the daily clinical practice of endocrinology. The increasing use of US and other imaging techniques has led to an epidemic of nodular lesions accompanied by a parallel increase in the diagnosis of thyroid cancers in almost all developed countries [14, 15]. Thus, there is a need for guidelines to help the clinician to manage a disease that is often asymptomatic and rarely lethal. Efforts have been made to refine the US neck examination to limit the FNA of nodules. For several years, the AACE has been involved in the education and certification of neck US (ECNU), while AME began a similar certification in 2016 and the European Thyroid Association developed guidelines about US risk stratification in 2017. In this paper, we attempted to verify the usefulness of the suggested US risk categories proposed by the AACE/AME and the indication for FNA in clinical practice.

Our results showed that, as expected, thyroid nodules mostly affect middle aged women and are usually benign and ovoid-to-round shaped, and the greatest dimension is about 20 mm (average). In nodules evaluated by FNA, the dimensions tended to inversely correlate with the US risk category, with larger nodules having a lower US risk of malignancy. These data confirmed that the selection criteria for FNA reasonably direct the clinician to biopsy small lesions when suspicious features are detected, while leaving out greater lesions that appear benign. Indeed, in about 1/3 of our cases, high risk nodules were <10 mm. These data are consistent with the findings that 39% of papillary thyroid cancers diagnosed in the United States in 2008-2009 were <10 mm, and the increased rate of papillary cancer is mostly due to small tumors [14, 15]. Clearly, our clinical practice is more likely to perform biopsy rather than just monitor.

In this cohort of patients, the overwhelming majority of low risk nodules had a diameter between 10 and 20 mm or >20 mm. This is the most strident difference between our results and the AACE/AME guidelines that generally suggest avoiding FNA in such cases. Of note, low risk nodules represented about 40% of all the nodules evaluated by FNA in our cohort. The following reasons may explain the elevated number of biopsied low risk nodules. (1) In our Division of Endocrinology, large benign nodules often undergo laser ablation and this procedure requires cytologic confirmation that the nodules are benign. (2) We usually try to avoid surgery, when possible; some examples of this include a case with an autonomously functioning nodule that was a candidate for radioactive treatment, a case with a cystic lesion that was a candidate for percutaneous ethanol injection, and a case with a large benign nodule that was a candidate for laser ablation, but concomitant nodules were biopsied anyway. (3) We also biopsy nodules for patients referred to our division from other hospitals; thus, we do not always make the biopsy decision. Even when considering all these reasons, it is clear that FNA is commonly overused, often to reassure the patient. In addition, nodules <20 mm that have an intermediate risk represented about 40% of all our nodules evaluated by FNA. The AACE/AME guidelines also discourage FNA for these cases. The decision to biopsy intermediate risk nodules often involves their overall management. In most cases, the patient opts for FNA instead of US monitoring to be assured the lesion is benign; then, US can be used to monitor at 2-3-year intervals [16–19]. In contrast, knowing that a <20 mm intermediate nodule is malignant or at risk of malignancy would allow the planning of a surgical strategy, instead of monitoring of the nodule yearly until it becomes >20 mm. A more aggressive diagnostic approach than suggested by current guidelines was also present in Italian and North American surveys [20, 21].

The clinical usefulness of AACE/AME US risk stratification was confirmed by the cytologic reports. In low risk nodules, just 1% (once excluded inadequate samples) had a moderate-to-elevated risk of malignancy or was malignant (TIR3B/TIR4/TIR5). This rate increased to 4% for the intermediate risk nodules and reached 84.6% for the high risk ones. In our cohort of patients, four (15.4%) high risk nodules had an absent or low risk of malignancy (TIR2/TIR3A). Of these patients, a benign nodule (TIR2) was confirmed in one patient by a second FNA, the final histologic exam demonstrated there was papillary cancer in two patients whose cytologic report identified the nodule as TIR3A, and one patient was lost to follow-up. These data confirm that even if cytology finds a low risk of malignancy, US features of malignancy should always be considered in a physician's decision-making. The most difficult problem to deal with in the management of thyroid nodules is clearly represented by the intermediate risk class, as the number of malignant or suspicious as malignant nodules is not negligible. In our series, limiting FNA to intermediate risk nodules >20 mm would have missed 13/17 (76%) nodules that had a moderate-to-elevated risk of malignancy or were malignant (11/13 were malignant by histology). The AACE/AME guidelines state that the expected risk of malignancy in intermediate risk nodules is 5–15%. The expected risk of malignancy reported by the guidelines refers to the US features of the nodule itself, independent of its diameter; in other words, the same guidelines that established a 20 mm diameter threshold for FNA implicitly accept that 5–15% of intermediate risk nodules <20 mm may be malignant. In our cohort of patients, 3.9% had a moderate-to-elevated risk of malignancy or were malignant by cytology; of these, 75% had a diameter <20 mm. In our series, 69% of intermediate risk nodules had a diameter <20 mm; therefore, this result is not surprising.

There is unavoidable bias because the patients referred for FNA have already been selected. In other words, we cannot state that in an unselected population 75% of indeterminate nodules with suspicious cytologic features measured <20 mm. Our data confirmed, as stated by AACE/AME guidelines, that the risk of malignancy is about 5% for intermediate risk nodules despite their size. It is plausible that when following these guidelines, the diagnosis of malignancy is delayed in a nonnegligible number of patients if FNA is not done until the nodule is ≥20 mm. Given that the mortality rate is not reduced if there is cytologic assessment of intermediate risk nodules <20 mm, previous findings strongly support that an earlier diagnosis is associated with less lymph node involvement, extrathyroidal extension, and lower recurrence rates [22–24].

In conclusion, the US risk categories suggested by AACE/AME guidelines and the relative suggestions for FNA perform well for low and high risk nodules, although, in clinical practice, small high risk nodules are more frequently biopsied than just monitored. The need for FNA in intermediate risk nodules <20 mm is still a matter of debate, given the nonnegligible risk of malignancy or suspicion for malignancy. Our results also suggest that the biopsy decision be patient-oriented (taking into account the clinical setting, operator expertise, and the patient's preference) rather than be based on nodule-size.

## Figures and Tables

**Table 1:** Dimensions of nodules evaluated by fine needle aspiration, divided by ultrasound risk categories. Data are expressed as the mean ± SD.

	Low risk	Intermediate risk	High risk	*P*
	(341)	(489)	(29)	
Anteroposterior (mm)	15 ± 7.8	14.0 ± 7.4	10.9 ± 8.0	Low risk versus high risk < 0.1
				Low risk versus intermediate risk = NS
	(5–59)	(4–49)	(5–36)	
				Intermediate risk versus high risk = NS

Transverse (mm)	14.8 ± 6.2	14.0 ± 6.2	11.0 ± 7.6	Low risk versus high risk = <0.01
				Low risk versus intermediate risk = NS
	(4–40)	(4–42)	(5–35)	
				Intermediate risk versus high risk = 0.01

Longitudinal (mm)	21.0 ± 9.9	18.8 ± 8.8	15 ± 13	Low risk versus high risk < 0.01
				Low risk versus intermediate risk < 0.01
	(7–80)	(6–65)	(5–56)	
				Intermediate risk versus high risk < 0.05

Volume (mL)	4.1 ± 7.8	3.4 ± 6.4	3.3 ± 9.0	Low risk versus high risk = NS
				Low risk versus intermediate risk = NS
	(0.1–93.3)	(0.1–64.2)	(0.1–35.7)	
				Intermediate risk versus high risk = NS

**Table 2:** The greater diameters of thyroid nodules evaluated by fine needle aspiration, divided by ultrasound risk categories. Data are expressed as the mean ± SD.

	<10 mm	10–20 mm	>20 mm	*P*
Low risk (341)	18 (5.3%)	164 (48.1%)	159 (46.6%)	<10 versus 10–20 < 0.01
				<10 versus >20 < 0.01
				10–20 versus >20 = ns

Intermediate risk (489)	41 (8.3%)	295 (60.4%)	153 (31.3%)	<10 versus 10–20 < 0.01
				<10 versus >20 < 0.01
				10–20 versus >20 < 0.01

High risk (29)	11 (37.0%)	13 (44.5%)	5 (18.5%)	<10 versus 10–20 = NS
				<10 versus >20 = NS
				10–20 versus >20 = NS

*P*	Low versus high risk < 0.01	Low versus high risk = NS	Low versus high risk < 0.05	
	Low versus intermediate risk = NS	Low versus intermediate risk = NS	Low versus intermediate risk < 0.01	
	Intermediate versus high risk < 0.01	Intermediate versus high risk = NS	Intermediate versus high risk = NS	

**Table 3:** Distribution of cytologic results according to the ultrasound risk categories of nodules evaluated by fine needle aspiration.

	TIR1	TIR2	TIR3A	TIR3B	TIR4	TIR5	*P*
Low risk (341)	29	276	33	3	0	0	TIR2 versus others < 0.01
	(8.5%)	(80.9%)	(9.7%)	(0.9%)	(0%)	(0%)	

Intermediate risk (489)	51	328	93	11	4	2	TIR2 versus others < 0.01
	(10.4%)	(67.1%)	(19%)	(2.3)	(0.8%)	(0.4%)	

High risk (29)	3	1	3	5	2	15	TIR5 versus others < 0.01
	(10.3%)	(3.5%)	(10.3%)	(17.3%)	(6.9%)	(51.7%)	

*P*	NS	Low versus intermediate risk = NS	Low versus intermediate risk < 0.01	Low versus intermediate risk = NS	Low versus intermediate risk = NS	Low versus intermediate risk = NS	
		Low versus high risk < 0.01	Low versus high risk = NS	Low versus high risk < 0.01	Low versus high risk < 0.01	Low versus high risk < 0.01	
		Intermediate versus high risk < 0.01	Intermediate versus high risk = NS	Intermediate versus high risk < 0.01	Intermediate versus high risk < 0.05	Intermediate versus high risk < 0.01	
